# Dietary Supplements Containing Oat Beta-Glucan and/or Green Coffee (Poly)phenols Showed Limited Effect in Modulating Cardiometabolic Risk Biomarkers in Overweight/Obese Patients without a Lifestyle Intervention

**DOI:** 10.3390/nu15092223

**Published:** 2023-05-08

**Authors:** Joaquín García-Cordero, Raquel Mateos, Susana González-Rámila, Miguel A. Seguido, José Luis Sierra-Cinos, Beatriz Sarriá, Laura Bravo

**Affiliations:** 1 Department of Metabolism and Nutrition, Institute of Food Science, Technology and Nutrition (ICTAN), Spanish National Research Council (CSIC), C/Jose Antonio Novais 10, 28040 Madrid, Spain; 2 Department of Nutrition and Food Science I, School of Pharmacy, Universidad Complutense de Madrid, Ciudad Universitaria s/n, 28040 Madrid, Spain

**Keywords:** oat beta-glucan, green coffee hydroxycinnamates, obesity, lipid metabolism, glucose homeostasis

## Abstract

Obesity has reached pandemic proportions and has become a major health concern worldwide. Therefore, it is necessary to find new strategies against this condition and its associated comorbidities. Green coffee polyphenols (GCP) and oat beta-glucans (BGs) have proven their hypolipidaemic and hypoglycaemic effects. This study aimed to examine the effects of the long-term consumption of supplements containing GCP, BG or the novel GCP/BG combination on lipid and glucose metabolism biomarkers in overweight/obese subjects who maintained their dietary habits and physical activity, hence addressing the difficulty that this population faces in adapting to lifestyle changes. A randomised, crossover, blind trial was carried out in 29 volunteers who consumed either GCP (300 mg), BG (2.5 g) or GCP/BG (300 mg + 2.5 g) twice a day for 8 weeks. Blood samples were collected, and blood pressure and body composition were measured at the beginning and end of each intervention. Total cholesterol, triglycerides, high-density lipoprotein (HDL-C), low-density lipoprotein (LDL-C), very low-density lipoprotein (VLDL-C) cholesterol, glycated haemoglobin, fasting glucose, insulin, aspartate transaminase, alanine transaminase and different hormones and adipokines were analysed. Only VLDL-C (*p* = 0.01) and diastolic blood pressure (*p* = 0.027) decreased after the intervention, especially with the BG supplement. There were no other significant changes in the analysed biomarkers. In conclusion, the regular intake of GCP, BG and GCP/BG without lifestyle changes is not an efficient strategy to improve lipid and glucose homeostasis in overweight/obese subjects.

## 1. Introduction

In recent years, overweight/obesity has become a major health concern worldwide, with the latest World Health Organisation (WHO) report stating that, in Europe alone, approximately 60% of adults suffer from this condition [1]. Many non-communicable diseases, including cardiovascular diseases (CVD) and certain types of neoplasms, share overweight/obesity as a major risk factor for their development. Excess body weight is a key factor in the condition known as metabolic syndrome (MS), defined by the WHO as the combination of several known cardiovascular risk factors, including abdominal obesity, hyperlipidaemia, insulin resistance and hypertension [2]. The incidence of MS often parallels that of obesity [2] and type 2 diabetes mellitus (T2DM), the incidence of which in Europe has doubled since 1980 (from 4.7% to 8.5% in 2014) [3]. With the dramatic increase in the prevalence of overweight/obesity and the difficulties that the affected population faces in making long-term changes to dietary habits and physical activity, it has become clear that there is a strong need for complementary strategies to combat both overweight/obesity and associated cardiometabolic traits such as hyperlipaemia, insulin resistance, etc. Nutraceuticals or dietary supplements are postulated as candidate tools to combat obesity and its associated comorbidities thanks to their remarkable content of bioactive compounds with health benefits. Among nutraceuticals, those with a high content of soluble dietary fibre (SDF), such as pectins, gums, inulin or β-glucans, as well as plant-derived extracts rich in phenolic compounds, such as those obtained from green tea and coffee, yerba mate or guaraná, have attracted the attention of the scientific community due to their multiple beneficial properties [4,5]. 

Focusing on dietary fibre, both soluble and insoluble dietary fibre (IDF) promote digestion and contribute to weight loss by interfering with the absorption of fats and sugars and modulating gastric emptying, thus leading to an increase in satiety. However, only SDF has a prebiotic effect, being able to be metabolised by the colonic microbiota, promoting the growth of certain species, such as lactic acid bacteria and bifidobacteria [6]. As a form of SDF, β-glucans (BG) have gained special attention from the scientific community due to their physicochemical properties, especially their ability to form viscous solutions at low concentrations [7], delaying gastric emptying, slowing down the enzymatic breakdown of digestible carbohydrates such as starch, and decreasing the absorption of carbohydrates [5,8]. BG are fermented by the gut microbiota, increasing the proliferation of *Bacteroidota*, *Bacteroides*, *Prevotella*, *Bifidobacterium* and *Lacticaseibacillus* while reducing the counts of *Bacillota* and *Dorea* [9,10]. In addition, short-chain fatty acids (SCFAs) produced after BG fermentation are able to modulate the expression of the glucose transporter GLUT 4 in peripheral tissues (muscle and adipose tissues), promoting glucose uptake and reducing insulin resistance [11]. All this results in beneficial properties against obesity and hyperglycaemia, reducing postprandial glycaemic and insulinaemic responses to improve insulin sensitivity, as observed in both diabetic [12,13,14,15] and non-diabetic subjects [15,16,17]. Given this evidence, the European Food Safety Authority (EFSA) issued a favourable opinion regarding the consumption of BG, setting a recommended intake of 4 g of BG per 30 g of digestible carbohydrates [18]. 

In addition to their hypoglycaemic properties, BGs also have a marked hypolipidaemic effect, as observed in numerous clinical trials. Two major meta-analyses, one by Ho et al. [19] including a total of 58 randomised clinical trials on the lipid-lowering properties of oat BG supplementation and another by Llanaj et al. [20], which examined 59 randomised clinical trials on the cardiovascular-risk-factor-lowering capacity of oat supplementation, concluded that the consumption of oat BG decreased cardiovascular risk factors by reducing serum levels of total cholesterol (TC), triglycerides (TG), low-density lipoprotein cholesterol (LDL-C) and apolipoprotein B (Apo B) [19,20], together with an improvement in anthropometric parameters in patients with mild metabolic disturbances (obesity, hypercholesterolaemia) [20]. A recent meta-analysis of 13 trials on oat BG that included 927 hypercholesterolaemic participants also observed a significant decrease in TC and LDL-C, yet not in TG and high-density lipoprotein cholesterol (HDL-C), revealing that outcomes were critically affected by factors such as the disease severity of patients, the daily intervention with oat BG and its source, as well as the duration of the intervention [21]. However, there is controversy on the effects of oat BG on TG and HDL-C, which was specifically addressed in another recent systematic review. Of the 17 studies included in this review, 6 reported reductions in TG, and only 1 reported an improvement in HDL-C levels [22], concluding that more research on this topic is required.

The mounting evidence supporting the effect of BG in regulating cholesterol levels resulted in another favourable opinion by the EFSA regarding BG properties, stating that the regular consumption of at least 3 g/d of BG contributes to maintaining normal blood cholesterol levels [23], with a specific mention of the blood-cholesterol-lowering effect of oat BG [24]. This effect is also associated with its high viscosity, slowing down gastric emptying and hindering the action of digestive enzymes and bile salts, thus decreasing the absorption of fats and bile acids [7,25]. This reduction in bile acid absorption leads to a reduction in hepatic cholesterol levels and the increase of de novo synthesis of fatty acids via activation of enzymes such as 7-α-hydroxylase or 3-hydroxy-3-methylglutaryl coenzyme A reductase (HMGCoA-reductase), as well as the expression of LDL-C receptors [26]. In addition, SCFAs produced after BG fermentation are able to prevent hepatic cholesterol synthesis via inhibition of the enzyme acetyl-CoA carboxylase [27]. This hypolipidaemic effect, in addition to their ability to reduce carbohydrate absorption and postprandial glycaemic response, may also explain the hypertensive properties of BG, with studies on hypertensive subjects showing that the consumption of BG-rich foods such as oats significantly reduced systolic (SBP) and diastolic blood pressure (DBP) [28,29]. 

Concerning polyphenols, many studies have shown that these bioactive compounds exhibit a wide range of health-promoting properties. Among polyphenols, it is worth mentioning hydroxycinnamates, a large group of compounds (notably caffeic, ferulic, *p*-coumaric and chlorogenic acids) that are the major phenolic compounds in plant extracts obtained from green coffee, yerba mate, etc. [30,31,32]. Numerous clinical trials have shown the beneficial properties of hydroxycinnamates against overweight/obesity and hypoglycaemia, as reviewed by García-Cordero et al. [4]. Briefly, human studies have shown improvements in cardiovascular parameters, such as the lipid profile and plasma antioxidant capacity, following the consumption of foods rich in hydroxycinnamates, such as green coffee [33] or yerba mate [34], in hypercholesterolaemic subjects. These hypolipidaemic effects have been associated with the capacity of hydroxycinnamates to inhibit key enzymes involved in fatty acid synthesis in the liver, such as HMGCoA-reductase and acetyl-coenzyme A-acetyltransferase (ACAT), as well as to improve insulin and leptin sensitivity, reduce the expression of genes involved in lipogenesis, and increase β-lipid oxidation and peroxisome proliferator-activated receptor alpha (PPAR-α) expression in the liver [35]. In addition to their hypolipidaemic effects, hydroxycinnamates have also been shown to improve glucose homeostasis and insulin sensitivity, as observed in clinical studies with diabetic patients [36], healthy individuals [37] and subjects at risk of developing MS [38], among others. These hypoglycaemic effects are associated with the capacity of hydroxycinnamates to reduce glucose absorption in the gastrointestinal tract, increase glucose uptake in muscle and adipose tissues and enhance the expression of glucose transporter GLUT4 [35,39]. Moreover, hydroxycinnamates have been shown to improve satiety by increasing peptide YY (PYY) levels and stimulating the secretion of the incretin hormone glucagon-like peptide-1 (GLP-1) in the intestine [40,41] and exert a prebiotic effect by increasing the metabolic activity and count of *Bifidobacterium* spp. [42], which may support their hypolipidaemic and hypoglycaemic effects. 

Considering all the scientific evidence on the benefits of oat BG and hydroxycinnamates on overweight/obesity, we hypothesised that the combination of both bioactive compounds could have synergistic effects, thus providing a more reliable nutritional tool to combat this pathology. Therefore, the aim of the present study was to investigate the effects of the regular consumption of food supplements containing oat BG, hydroxycinnamates from a decaffeinated green coffee (GCP) extract or the combination of both on different lipid and glucose metabolic biomarkers in subjects with overweight/obesity.

## 2. Materials and Methods

### 2.1. Subjects and Ethical Considerations

**Figure 1:**
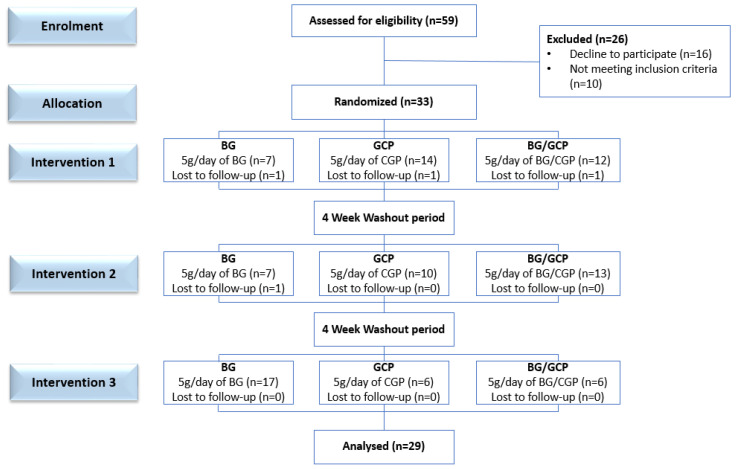
Study flow diagram (Consolidated Standards of Reporting Trials, CONSORT 2010).

For the present trial, the primary outcome established was a reduction in body weight. Changes in blood lipids, insulin resistance and body fat percentages were established as secondary outcomes. Based on this, the inclusion criteria for the recruitment of volunteers were as follows: men and women between 18 and 60 years old, with a body mass index (BMI) of 25–35 kg/m^2^. Although it was not established as a specific inclusion criterion, we aimed at recruiting participants with fasting glucose between 6.11 and 6.94 mmol/L (110–125 mg/dL) and/or glucose between 7.77 and 11.04 mmol/L (140–199 mg/dL) 2 h after an oral glucose overload as a surrogate indicator of the metabolic risk associated with overweight/obesity. Exclusion criteria were suffering from any chronic pathology other than overweight, obesity or pre-diabetes; smoking; vegetarianism; pregnancy/lactation in women; hypersensitivity or allergy to some of the ingredients of the nutraceuticals to be studied; and taking antibiotics, dietary supplements or hormones. Recruitment started in October 2018 and was carried out by placing advertisements at the campus of Universidad Complutense de Madrid, on the webpage of the Institute of Food Science, Technology and Nutrition (ICTAN-CSIC) and through social media. It is important to mention that it was intended to extend recruitment in order to reach the estimated sample size, but the SARS-CoV-2 pandemic made it impossible to enrol more volunteers or to extend the intervention. Out of 59 volunteers interviewed, 33 were admitted into the study and allocated to the experimental groups (Figure 1). Four participants were lost to follow-up, the rest of the volunteers completed the study. Of the 29 participants, 17 were male and 12 were female, aged between 28 and 59 years (average 45.2 ± 1.8). The intervention started in December 2018 and finished in November 2019. 

Approval of the study was obtained from the Clinical Research Ethics Committee of Hospital Universitario Puerta de Hierro, Majadahonda in Madrid (Spain), and the Bioethics Committee of Consejo Superior de Investigaciones Científicas (CSIC). It also followed the guidelines laid down in the Declaration of Helsinki for experiments in humans. Written informed consent was obtained from all volunteers before the start of the study. The study was registered in ClinicalTrials (NCT05009615). 

### 2.2. Food Supplements

Detailed information on the composition of the food supplements has been described elsewhere [43]. Briefly, 70% BG with molecular weight up to 200 kg/mol and a density of 0.5 g/mL was used (B-Can^TM^, Garuda International, Inc., Exeter, CA, USA). The GCP extract used, containing 45.8% hydroxycinnamic acids [43], was provided by Quimifarma Laboratorios S.L. (Toledo, Spain). Each individual sachet of the formulated nutraceutical contained 3.57 g of 70% BG and 0.66 mg of GCP, providing half the daily dose of bioactive compounds (2.5 g of the SDF in the BG supplement, 300 mg of phenolic compounds in the GCP nutraceutical, and the combined doses in the GCP/BG supplement). Volunteers consumed two sachets of the corresponding supplement per day, amounting to 5 g/d of BG and 600 mg/d of hydroxycinnamates. The doses were selected following the results of a previous 6-week, parallel, dose–response study carried out by our group in overweight/obese volunteers [43]. Sachets were labelled A, B or C for blinding, and each type of nutraceutical was offered with different aromas/flavours (chocolate, lemon and forest fruits) for better acceptance.

### 2.3. Study Design

The study was a randomised, crossover, three-arm, blind intervention that lasted 8.5 months. After a 2-week run-in, volunteers were randomly allocated to begin consuming GCP, BG or GCP/BG; randomisation was carried out using the Microsoft^®^ Excel 2016 program. Each intervention lasted 8 weeks and was separated by a 4-week wash-out period. The duration of the study was selected based on previous studies carried out by our research team with a green/roasted coffee blend [33,37,38] and also by other authors who studied oat products [44,45], and it was longer than most trials with BG, as reviewed in [19,20,21,22]. We aimed at gender parity when establishing the initial subgroups that started consuming the nutraceuticals. Volunteers consumed the food supplements corresponding to each intervention stage twice a day at different times of the day (half an hour before breakfast and before lunch) after dissolving the product in 250 mL of water. Compliance was controlled by weekly calls to the participants and by counting the number of returned, non-used sachets at the end of each stage.

At the beginning and end of each intervention period, volunteers attended the Human Nutrition Unit (HNU) at the Institute of Food Science, Technology and Nutrition (ICTAN). During the two days prior to each visit, participants were instructed to avoid caffeine and to consume a low-polyphenol diet, with special emphasis on the avoidance of foods rich in hydroxycinnamates, such as herbal teas; artichokes; red wine; vegetables; whole-grain cereals; and fruits, including dried fruits, juices and smoothies. Only watermelon, banana, potatoes and cantaloupes were allowed. In each visit, the subjects came to the HNU after an overnight fast. Then, a blood sample was obtained from the cubital vein by a licensed health care professional and collected into tubes without anticoagulant or in EDTA-coated tubes to obtain serum and plasma samples, respectively. In addition, blood pressure was measured, and anthropometric data were collected. SBP, DBP and heart rate were measured in triplicate in the non-prevailing arm with an OMRON^®^ M2 HEM-7121-E sphygmomanometer (OMRON HEALTHCARE Co., Ltd., Kyoto, Japan). Patients had to rest in the sitting position for 15 min to stabilise blood pressure before measurement, with pauses of 5 min between each measurement. Body weight, circumferences and skinfolds were measured following standard anthropometric protocols, and body composition was analysed using an INBODY S10 Body Composition Analyzer. Results on body weight and composition are reported elsewhere [46]. 

Participants were asked to refrain from changing their dietary and physical activity habits. Diet was monitored by collecting 72 h dietary recall questionnaires (which included two working days and one weekend day), which volunteers completed before each visit to the HNU. Energy and macronutrient and micronutrient intake were calculated using DIAL software for Windows (version 3.0.0.5; Department of Food Science and Nutrition, School of Pharmacy (UCM) and Alce Ingeniería, S.A. Madrid, Spain). 

### 2.4. Analysis of Metabolism Biomarkers

After collecting the blood samples, serum and plasma were obtained by centrifugation at 3000× *g* for 10 min, aliquoted and frozen at −80 °C until analysis. TC, TG, HDL-C, LDL-C, very low-density lipoprotein cholesterol (VLDL-C), glucose, glycated haemoglobin (HbA1c), insulin, aspartate transaminase (ASAT) and alanine transaminase (ALAT) were determined in serum samples following reference methods or methods recommended by Sociedad Española de Bioquímica Clínica y Patología Molecular (SEQC) using a Roche Cobas Integra 400 plus analyser (Roche Diagnostics, Mannheim, Germany).

C-peptide, ghrelin, gastric inhibitory polypeptide (GIP), glucagon-like peptide-1 (GLP-1), leptin, glucagon insulin, plasminogen activator inhibitor (PAI-1), resistin and visfatin concentrations were analysed in plasma using the Bio-Rad Multiplex Diabetes kit (Ref.: 171A7001M). The plasma samples were diluted 1:4 with a Bioplex sample diluent included with the kit prior to analysis. Adipsin and adiponectin concentrations were analysed using the Bio-Rad Multiplex Adipokine kit (Ref.: 171A7002M). In this case, a 1:2500 dilution with a serum-based diluent was employed according to the kit manufacturer’s instructions. In both cases, 50 µL of the diluted plasma sample was placed in each well of the assay plate in duplicate. The analytes were measured on a Bio-Plex MAGPIX™ Multiplex reader connected to a Bio-Plex ProTM Wash Station. The software Bio-Plex Manager™ MP (Luminex Corporation, Austin, TX, USA) was used for data processing. Results were expressed as pg/mL or ng/mL plasma.

### 2.5. Statistical Analysis

The sample size was calculated using the G*Power 3.1.9.7 program, considering body weight as the main variable. Assuming a statistical power of 80%, a level of statistical significance of 5%, a two-tailed hypothesis and a standard deviation of 6.5 and aiming to detect a difference of 2.5 kg, the sample size was calculated to be 38 subjects. 

The statistical analysis was performed using SPSS software (version 27.0; SPSS, Inc., IBM Company, Armonk, NY, USA). Values were expressed as mean ± standard deviation in the case of dietary data and mean ± standard error of the mean in the case of blood pressure and all the different biochemical and hormonal parameters. The level of statistical significance was set at *p* < 0.05. A preliminary Boxplot analysis was carried out in order to detect extreme values (outliers) and to determine the dispersion and symmetry of the data. In addition, the normality of the distribution and homogeneity of variance were evaluated using the Kolmogorov–Smirnov and Levene tests, respectively. 

Based on the results of the normality test, a general linear model of repeated measures was used to assess the evolution of the different parameters of energy, macronutrient and micronutrient intake at different time points throughout the study. The order of intake of the nutraceuticals did not matter for the purposes of the dietary analysis since it would not affect the overall dietary pattern of the volunteers. In contrast, with the other parameters, a linear mixed model was used, as this model allows the determination of the correlated variability and the study of the effects of the product consumed, taking into account the order of ingestion. For each variable studied, apart from statistically analysing the baseline values from the start of each intervention and the end values of each intervention, the rates of change were calculated [(end value − baseline value)/baseline value] to better estimate the effects produced by the treatment, evaluating how much the variable increased or decreased with respect to the baseline value. When statistical differences were observed, a Bonferroni post hoc test was used in order to conduct pairwise comparisons between the groups’ means. 

## 3. Results

The participants in the study were 17 men and 12 women with a mean BMI of 30.1 ± 0.6 kg/m^2^. There were no statistically significant changes in body weight or body fat percentage nor in any other anthropometric parameters, as reported by García-Cordero et al. [46].

### 3.1. Dietary Intake

The data on macro- and micronutrient intake during the study are shown in Table 1. There were no statistically significant differences at baseline or at the end of each intervention due to the intake of any of the food supplements (GCP, BG and GCP/BG) related to energy, macronutrient and micronutrient intake, showing that volunteers maintained their dietary habits for the duration of the study, as recommended. There was only a statistically significant change in iron intake, especially during the GCP intervention.

### 3.2. Cardiovascular Parameters

Table 2 shows the main results obtained in relation to the different parameters associated with lipid metabolism at baseline and at the end of the intervention, together with the corresponding rates of change. At the beginning of the study, volunteers’ blood TC levels were slightly high, so they presented mild hypercholesterolaemia considering the upper limit of normal blood cholesterol levels (200 mg/dL). Similarly, HDL-C levels were lower than the reference values (40–60 mg/dL), whilst LDL-C, TG and VLDL-C levels were within the healthy range. As can be seen, there were no statistically significant differences in the lipid profile between baseline values and after the intake of GCP, BG or GCP/BG, nor in their rates of change. There was only a significant difference in the rate of change in the serum levels of VLDL-C due to the consumption of BG and GCP, since BG alone or combined with GCP resulted in a reduction in VLDL-C, while the nutraceutical containing only GCP did not appear to modify this parameter. A tendency to improve blood lipids after BG or GCP/BG was also observed with TG, TC and HDL-C concentrations, although the differences did not reach statistical significance.

Table 3 shows blood pressure and heart rate results. Volunteers were normotensive (SBP < 140 mmHg, DBP < 90 mmHg). Interestingly, there was a statistically significant modification in the rates of change in DBP after the intervention, with BG again showing a significant reduction in DBP, while GCP showed little effect. 

### 3.3. Glucidic Metabolism

Table 4 shows the results obtained in relation to biochemical parameters associated with glucose metabolism at baseline and at the end of the interventions, together with the corresponding rates of change. At the beginning of the study, blood glucose levels and the percentage of HbA1c were not far from the limit considered indicative of pre-diabetes (100 mg/dL and 5.7%, respectively). As mentioned, pre-diabetes was not a specific inclusion criterion, and indeed, volunteers were below the limits established for this condition. We did not observe any significant changes in the values of HbA1c, glucose or insulin in serum nor in the rates of change in any of the parameters analysed after the intake of GC, BG or GC/BG.

### 3.4. Adipokine Hormone Analysis

Table 5 shows the main results of the adipsin and adiponectin analysis in serum samples before and after the intervention with each dietary supplement. No significant changes between the different treatments at the baseline, at the end of the intervention, or among the rates of change were obtained. 

Similarly, no significant changes were observed in other biomarkers shown in Table 6, such as C-peptide, ghrelin, GIP, GLP-1, glucagon, insulin, leptin, PAI, resistin and visfatin, before and after the intervention with each dietary supplement. 

### 3.5. Hepatic Function and Inflammatory Markers

Finally, Table 7 shows the concentrations of ASAT, ALAT and high-sensitivity C-reactive protein (hsPCR) obtained from serum samples before and after the intervention with each dietary supplement. No significant changes were observed in the rates of change after consuming any of the different food supplements. 

## 4. Discussion

Changes in dietary habits, such as reducing total energy intake, limiting the consumption of fat- and carbohydrate-rich foods and increasing the consumption of plant-based foods such as vegetables, legumes, fruits and nuts, along with an active lifestyle and regular exercise, are considered the most effective strategies to reduce the incidence of overweight and obesity and to prevent their associated comorbidities [1]. However, this population with excess body weight is often characterised by having problems in achieving these lifestyle changes in the long term, with a very high dropout rate from treatments restricting energy intake and increasing regular physical exercise. The number of patients with good adherence to hypocaloric diets has been estimated to be lower than 50%; in turn, 8 out of every 10 patients abandon full nutritional treatment before completion [47]. Considering that the alarming rates of overweight/obesity and associated non-communicable diseases are escalating worldwide, and that supportive policies carried out by governments at different levels (socio-sanitary, environmental, educational) have proven to be insufficient to promote healthier lifestyles, it is necessary to find alternative strategies to combat these pathologies. In this context, the aim of the present work was to test the effectiveness of food supplements with potential hypolipidaemic and hypoglycaemic properties in the treatment of overweight/obesity and their comorbidities, such as insulin resistance, hypercholesterolaemia and hypertension. Positive results in this regard could lead to new dietary products, especially interesting for patients who have difficulty adhering to restrictive dietary treatments or increasing physical exercise to lose weight. 

According to the present data obtained from 72 h questionnaires, the intake of energy and nutrients did not change during the intervention (Table 1), confirming that volunteers maintained their previous dietary habits as established in the study protocol. Similarly, the food supplements seemed to have no effect on food intake, which is in agreement with the limited impact on appetite and satiety reported before for these nutraceuticals [40]. Dietary recommendations for the Spanish population establish that lipids and proteins should provide 30–35% and 10–15% of the daily caloric intake, respectively [48]. However, according to the results in Table 1, proteins provided up to 17.3% of the energy, and lipids contributed over 40%, with a higher consumption of saturated fatty acids than that established in dietary recommendations, showing that volunteers did not maintain a balanced diet. This is in accordance with the reported changes in the dietary habits of the Spanish population, whose adherence to the Mediterranean diet has been decreasing in the last few decades [49].

Strong scientific evidence supports that regular BG consumption improves health by maintaining normal blood cholesterol and glucose levels, as demonstrated by the positive opinions issued by the EFSA [18,23,24]. Similarly, there is clinical evidence on the positive effects of hydroxycinnamate-rich foods, such as green coffee and yerba mate, in managing lipid metabolism and improving cardiovascular parameters and glucose homeostasis [33,34,35,36,37,38]. However, in the present trial, the consumption of 5 g/d of oat BG, 600 mg/d of green coffee hydroxycinnamates or the combination of both bioactive compounds for 8 weeks did not induce changes in the end values or rates of change in TC, TG, HDL-C and LDL-C. We observed significant differences in the rates of change in VLDL-C levels, with the food supplement containing GCP/BG being the one that reduced the levels of VLDL-C most efficiently, followed by the supplement containing only BG (Table 2). As for the other analysed cardiometabolic biomarkers related to blood pressure (Table 3) and glucose metabolism (HbA1c, glucose, insulin and different hormones) (Table 4 and Table 5), they were not affected by the intervention with any of the food supplements.

Changes in VLDL-C were observed in a randomised clinical trial conducted by Reyna-Villasmil et al. [44] in 38 male subjects with mild to moderate hypercholesterolaemia, with supplementation of 6 g/day of oat BG added to the American Heart Association (AHA) Step II diet along with moderate physical activity for 8 weeks. Similarly, in a 6-week parallel, randomised, blind study carried out by our team in 60 volunteers, two doses (3 g/d or 5 g/d) of two types of oat BG (35% and 70% BG) were used together with a fixed amount of 600 mg of decaffeinated green coffee hydroxycinnamates [43]. After the 6-week intervention, the concentrations of TC, LDL-C, VLDL-C and TG significantly decreased compared to baseline, with all the food supplements being equally effective. These and previous antecedents supported the hypothesis that BG and GCP would positively affect the management of comorbidities associated with excess body weight.

In a recent systematic review and meta-analysis of 58 randomised clinical trials (*n* = 3971) in healthy and hypercholesterolaemic participants, Ho et al. [19] summarised the evidence showing that oat BG decreases LDL-C levels. In another review and meta-analysis by Llanaj et al. [20] including 59 randomised clinical trials (*n* = 4937) in predominantly hypercholesterolaemic and overweight subjects with mild metabolic disturbances, the consumption of oat BG was also shown to improve TC, LDL-C and glucose levels compared to controls. These authors also found inconsistent results for glucose homeostasis markers such as HbA1c and insulin concentrations. However, the effects of BG on TG and HDL-C are controversial, with only 6 out of 17 studies reporting the positive effects of oats or oat BG in reducing TG concentrations and 1 study observing HDL-C improvement after the consumption of oats, as recently reviewed by Amerizadeh et al. [22]. These authors found that oat intake might reduce TG in healthy people with normal blood lipids, as well as in overweight volunteers or people with diabetes or MS when consuming higher amounts of oats and/or during longer interventions [22]. Besides the dose of oats/oat BG consumed and the duration of the intervention, the systematic review and meta-analysis by Yu et al. [21] also revealed that the source of BG and the disease severity of the participants were factors with important impacts on the lipid-lowering effects of BG.

Another important factor affecting the potential outcomes of an intervention with oats/oat BG is their intake in conjunction with a calorie-restricted diet, which proved to be more effective [20,22]. Unlike in our trial, where volunteers’ diet and physical activity remained unchanged, in many studies, dietary intervention with caloric reduction is carried out in conjunction with BG supplementation. Thus, Charlton et al. [50] carried out a randomised controlled trial in 87 mildly hypercholesterolaemic and overweight men and women who were assigned for 6 weeks to one of three diets supplemented with cereals enriched with BG: minimal BG (control); low-dose oat BG (1.5 g BG; oats low—OL); or high-dose oat BG (3 g BG; oats high—OH). All volunteers followed a healthy low-fat diet with energy intakes calculated to adjust to the estimated energy requirements for weight maintenance. After the intervention, TC and LDL-C were reduced significantly in all groups, with higher reductions observed in the OL and OH diets compared to the control group [50]. In the parallel trial carried out by Reyna-Villasmil et al. [44], the consumption of 6 g of BG from oats added to the AHA Step II diet, combined with moderate physical activity, improved lipid profiles, although with different responses. Thus, HDL-C concentrations increased in volunteers on the AHA Step II diet that included whole-wheat bread (group A) but not in volunteers on the AHA Step II diet with bread containing 6 g of BG (Nutrim-OB, group B). In turn, LDL-C decreased in both groups, but more efficiently in group A than in the volunteers who consumed BG-enriched bread (27.89% A vs. 16.8% B), suggesting a more limited effect of BG [44]. 

Other studies also showed discrepancies in the effect of BG in improving the lipid profile or glucose homeostasis. In a parallel, controlled, 12-week study carried out by Adamsson et al. [51] in 79 overweight subjects consuming a normal breakfast (control group) or a low-fat breakfast with 40 g of oat bran porridge or muesli (corresponding to 3 g/d oat BG), no differences were found in LDL-C, TC, HDL-C, TG, the LDLC/HDL-C ratio, Apo A1, Apo B, glucose tolerance, HbA1c or insulin sensitivity. Similarly, in another parallel, placebo-controlled trial carried out by Biörklund et al. [52] in 43 healthy men and women with high TC levels, the daily consumption of 4 g of oat BG incorporated into a healthy ready meal for 5 weeks did not modify TC and LDL-C concentrations compared with the same ready meal without BG. These authors did not find any changes in postprandial glucose or insulin concentrations after the intervention [52]. The results of these two studies are in line with those obtained in the present crossover trial, where lipid levels (Table 2) or glucose homeostasis (Table 4) were not significantly modified after the intervention with any of the supplements. More recently, in a randomised, placebo-controlled trial assessing the effects of the intake of 3 g/day of oat BG in 83 Italian men and women with moderate consumption of a Mediterranean diet, significant reductions in LDL-C (12.2% and 15.1% from baseline), TC (6.5% and 8.9%) and non-HDL-C (11.8% and 12.1%) were observed after 4 and 8 weeks of treatment, respectively. However, there were no effects on blood pressure, fasting blood glucose or anthropometric parameters after the intervention [53]. Interestingly, although there were differences in the rate of change after 4 and 8 weeks of treatment, this study showed that even a short treatment (4 weeks) was effective in improving blood lipids in the hypercholesterolaemic subjects. 

Similar to that described for blood lipids, the effect of BG on glucose homeostasis also shows contradictory results. A recent systematic review on the influence of oat intake on CVD risk markers did not find convincing evidence of oats’ influence on insulin sensitivity since no studies described any significant effects on insulin sensitivity after long-term oat consumption [54]. Shen et al. [55] carried out a meta-analysis of randomised, controlled trials of four studies (*n* = 350) with T2DM patients and reached the same conclusions. These authors found that the administration of oat BG in doses from 2.5 to 3.5 g/day for 3 to 8 weeks significantly lowered fasting plasma glucose concentrations by −0.52% and HbA1c by −0.21% compared to controls, but not the levels of fasting plasma insulin [55]. Recently, Zurbau et al. [56] performed a systematic review and meta-analysis of 103 (*n* = 538) crossover, controlled trials investigating the effect of oat BG in carbohydrate-containing test meals compared to carbohydrate-matched BG-free control meals in humans, regardless of health status. These authors found that oat BG reduced the glucose incremental area under the curve (iAUC) and incremental peak rise (iPeak) by 23% and 28%, respectively, as well as a reduction in insulin iAUC and iPeak of 23% and 28%, respectively [56]. All these findings suggest that BG’s ability to improve glucose levels and, especially, insulin levels and insulin sensitivity is not fully clarified, in line with the results obtained in the present intervention. However, it is important to highlight that the BG dose, BG molecular weight and comparator were significant effect modifiers of glucose iAUC and iPeak in the meta-analysis by Zurbau et al. [56], with oat BG with a molecular weight > 300 kg/mol being the one able to significantly reduce glucose iAUC and iPeak. This could explain why, in our intervention, we did not find significant differences in any of the parameters analysed related to glucidic metabolism, as the oat BG employed had a lower molecular weight (between 100 and 200 kg/mol) [43].

As for hydroxycinnamates in the decaffeinated green coffee phenolic extract, the literature also shows controversial results on their effects in regulating blood lipids and glucose homeostasis. Thus, in a randomised, crossover, controlled study carried out in 25 normocholesterolaemic and 27 hypercholesterolaemic subjects, the consumption of 6 g/day of a soluble green/roasted (35:65) coffee blend (containing 74.2 mg/g of total hydroxycinnamic acids and 20.2 mg/g of total methylxanthines) for 8 weeks statistically reduced blood levels of TC, LDL-C, VLDL-C and TG in the hypercholesterolaemic group compared to the control drink [33]. In this study, significant improvements in plasma antioxidant capacity, SBP, DBP, heart rate and body weight were also observed in both normo- and hypercholesterolaemic volunteers, pointing to the remarkable positive health effects of the green/roasted coffee blend. In another randomised, controlled trial conducted by De Morais et al. [34] in 15 normolipidaemic, 57 dyslipidaemic and 30 hypercholesterolaemic subjects on long-term statin therapy, the consumption of 330 mL of green (5.51 ± 0.2 mg/mL of polyphenols) or roasted (1.74 ± 0.1 mg/mL of polyphenols) yerba mate infusions three times per day for 40 days increased HDL-C and decreased LDL-C, non-HDL-C, Apo B and SBP compared to controls, again showing the beneficial effects of this hydroxycinnamate-rich beverage. However, in a recent systematic review on the effects of food supplements containing hydroxycinnamic acids on cardiometabolic biomarkers, only nine out of eighteen studies showed a significant reduction in LDL-C and TC [57]. In a randomised clinical trial carried out in 43 subjects with MS who consumed 400 mg of green coffee extracts (186 mg of chlorogenic acids per capsule) twice per day for 8 weeks, in spite of significant reductions in SBP, fasting blood glucose, insulin resistance, waist circumference and subjective appetite compared to the placebo group, the authors did not find any statistically significant differences in HbA1c and lipid profile parameters [58]. This is in line with the results reported here for GCP, providing 600 mg/d of hydroxycinnamic acids, since this food supplement, alone or in combination with BG, did not produce changes in any lipid and glucose metabolism biomarkers.

Furthermore, Pourmasoumi et al. [59], in a recent systematic review and meta-analysis of 15 (*n* = 637) clinical trials that examined the effect of green coffee bean extracts on cardiometabolic risk factors, found that the regular intake of green coffee polyphenols significantly reduced the levels of fasting plasma glucose along with other cardiovascular biomarkers, such as SBP, DBP, TC and BMI. Subgroup analysis suggested more effective results with doses over 400 mg/day for more than 60 days of regular intake [59]. This is in contrast to the results reported here, as the consumption of 600 mg/day of green coffee polyphenols for 56 days (8 weeks) did not induce changes in any biomarkers related to glucose homeostasis. However, not all the studies found a significant improvement in glucose metabolism after treatment with hydroxycinnamate-rich foods. Interestingly, in a randomised, double-blind, placebo-controlled clinical trial carried out in 64 obese women, after treatment with 400 mg/day of a green coffee bean extract (180 mg of chlorogenic acid) combined with energy restriction for 8 weeks, there was a significant reduction in body weight, body mass and fat mass indices, TC, LDL-C, plasma free fatty acids, leptin and adiponectin compared to the placebo, but not in fasting blood glucose and insulin levels [60]. Additionally, the systematic review and meta-analysis of interventional studies carried out by Nikpayam et al. [61] concluded that there was not enough evidence supporting the effects of green coffee supplementation on insulin levels. 

Importantly, as with BG [21], different authors have highlighted that the health status of volunteers might be a potential modifier, as individuals with higher baseline cholesterol, blood pressure, glucose levels or insulin resistance showed greater improvements after treatment with foods or supplements containing hydroxycinnamic acids [57,58,59]. Participants in the present study had only marginally high TC levels (<207 mg/dL, Table 2), were normotensive (Table 3) and had normal fasting blood glucose concentrations at baseline (93.7–95.6 mg/dL, Table 4).

In relation to other cardiovascular biomarkers, we found a significant difference in the rate of change in DBP between food supplements, with a higher reduction in blood pressure levels with the BG supplement compared to GCP. This result is in agreement with previous studies, especially with a recent systematic review and meta-analysis that observed significant reductions in DBP after supplementation with oats rich in BG [20]. However, this significant reduction was only observed in studies that combined oat supplementation with a dietary caloric restriction, which could explain why we did not observe significant differences at the end of the interventions. 

The concept of creating a food supplement that combines BG and GCP emerged from the idea that these compounds could exert complementary or synergistic actions, taking into account that polyphenols and SDF have different mechanisms of action (reviewed in [4]). In a recent study carried out by our group [40], the regular consumption of GCP/BG increased postprandial satiety, which was associated with increased leptin levels. Additionally, lower subjective appetite scores in the long term (after 8 weeks) were associated with lower ghrelin levels. In contrast, these appetite/satiating effects were not observed in the intervention with GCP in the same group of volunteers, pointing to the well-known effect of SDF (BG) on satiety [4,5,18]. Another reason for studying GCP/BG was that, as far as we know, there are no food supplements on the market that combine GCP and SDF. However, the regular consumption of GCP/BG did not show any improvements in lipid and glucose metabolism biomarkers, in contrast to those observed in a previous dose–response study with supplements containing two doses (3 and 5 g/d) of 35% and 70% oat BG combined with 600 mg/d GCP [43]. In this study, all the assayed biomarkers, including blood lipids, blood pressure, glucose homeostasis and body weight and composition, were improved with the four tested supplements after a 6-week intervention in 60 overweight/obese volunteers [43]. In that parallel study, according to the general model of variance with repeated-measures analysis, there were no differences among the treatments except for SBP and total body fat percentage. Looking into the rates of change in these two parameters, 5g-70% BG was the treatment that lowered total body fat percentage the most, and thus, it was the type and dose of BG selected for the present crossover intervention, since it was considered to be more effective in helping to lose weight.

Although not in humans, a recent study addressed the potential synergistic effect of a combination of oat phenolic compounds (OPCs), comprising mainly hydroxycinnamic acids and flavonoids, and oat BG (OBG) in mice fed a high-fat diet [62]. Both OPCs and OBG were able to regulate body weight, lower fasting blood glucose and improve blood lipids while also decreasing hepatic fat deposition after 8 weeks. Interestingly, the combination of OPC + OBG showed better effects, demonstrating the synergistic action of both bioactive fractions. The results of this animal study show that our initial hypothesis was not futile despite the lack of results after the three-arm intervention reported herein.

As mentioned, there are important factors that might account for the lack of significant effects on the different biomarkers assayed in the present study. Firstly, the biological activity of BG and hydroxycinnamate supplements highly depends on the source of the bioactive compounds and the extraction and purification methods used to obtain them [63]. The 70% oat BG used in this study had a high concentration of BG but a low molecular weight. This affected its viscosity, which was only 0.05 Pa·s^−1^ [43]. Although the molecular weight of barley BG was reported to have no impact on perceived feelings of hunger or on energy intake in healthy subjects [64], it has been reported that the consumption of high-molecular-weight but not low-molecular-weight barley BG reduced TC concentrations in mildly hypercholesterolaemic adults [65]. Similarly, positive effects of oat BG on reducing glucose iAUC and iPeak occurred with high-molecular-weight (>300 kg/mol) but not with <300 kg/mol BG [56]. The doses of BG (5 g/d) and GCP (600 mg/d) were selected according to EFSA scientific opinions substantiating the health effects of BG [18,23,24] and previous results with hydroxycinnamate-rich green coffee [33,37,38,59], and the duration of the intervention (8 weeks with each supplement) can also be considered sufficient in light of the existing evidence from studies of similar or even shorter duration [19,20,21,22,34,50,52,53,55,58,60], although longer-term studies are always advisable. As mentioned, the status of the volunteers at baseline pointed to an a priori healthy condition, since apart from overweight/obesity, they were normotensive, with normal fasting blood glucose and only marginally elevated TC levels. As highlighted in different systematic reviews and meta-analyses, more positive results with foods or supplements containing BG [19,20,21,22] or hydroxycinnamic acids [57,59] are observed in subjects with poorer basal conditions (individuals with higher TC, blood pressure or fasting glucose levels). However, the main limitation of the present study was the fact that the study was underpowered. The number of volunteers required was not reached in the first phase of the study, and four volunteers were lost during the intervention. Moreover, the outbreak of the SARS-CoV-2 pandemic impeded further recruitment and forced the intervention to finish with a small number of volunteers, so the question remains as to whether the formulated supplements might have been as effective as they proved to be in the previous dose–response intervention [43]. This study has some other limitations, such as the fact that real exposure and differences in the bioavailability and metabolism of green coffee phenolic compounds were not monitored, which might account for the observed different responses, since it is well known that interindividual variability in the bioavailability of phenolic compounds will greatly affect their biological activity [66]. Similarly, adherence was not controlled by measuring an objective biomarker of GCP intake, such as ferulic acid-4′-sulfate, dihydrocaffeic acid-3-sulfate or feruloylglycine in urine [67]. Another important point to take into account is the influence of the gut microbiota, as it is well established that overweight/obesity is associated with an altered microbiota, and it plays an important role in the status of the disease and the progression of MS and insulin resistance [9]. Considering the importance of microbiota dysbiosis in overweight/obesity as well as its crucial role in the metabolism of phenolic compounds, addressing the potential changes in the microbiota would have added complementary information and could help to explain the lack of results observed in the present study. 

Finally, it is important to highlight that the study design did not contemplate any lifestyle interventions, so volunteers were not under a dietary caloric restriction or increased physical activity regime. These are the main recommendations for weight reduction and the prevention of cardiometabolic diseases, and therefore, the rationale of a study that did not contemplate this approach might be considered inadequate. However, the lack of long-term adherence of obese subjects to these lifestyle changes prompted us to assess the potential effectiveness of consuming a dietary supplement without modifications in energy intake and expenditure. It would be certainly of interest to assess the potential effects of the studied nutraceuticals as adjuvants to healthy lifestyle modifications.

## 5. Conclusions

The regular intake of the studied food supplements containing BG could improve DBP and VLDL-C blood concentrations, which are of interest in cardiovascular health. However, the consumption of GCP, BG and a combination of both bioactive ingredients was not enough to induce significant positive changes in lipid and glucose metabolism biomarkers in overweight or obese people without changes in dietary and physical activity habits. However, we do not dismiss the possibility that if the study had been carried out in a larger group of volunteers, and/or if the interventions had been longer and in conjunction with lifestyle modifications, positive effects would have been observed, considering the health properties of BG and GCP.

## Figures and Tables

**Table 1:** Energy, macronutrient and micronutrient intake recorded in 72 h recalls filled in by volunteers at each intervention stage with the food supplements containing green coffee polyphenols (GCP), beta-glucan (BG) and the mixture of GCP/BG.

	BG		GCP		GCP/BG		*p*
	Baseline	End	Baseline	End	Baseline	End	
Energy (Kcal)	2058 ± 589	1937 ± 433	2019 ± 538	2115 ± 634	2024 ± 602.6	2128 ± 619.1	0.177
Protein (g)	84.8 ± 25.2	85.2 ± 22.3	82.8 ± 21.8	82.8 ± 25.1	79.7 ± 19.2	86.9 ± 24.2	0.443
CHO (g)	211 ± 70	196 ± 64	199 ± 60	204 ± 61	205 ± 76.9	200 ± 70.3	0.564
Dietary Fibre (g)	23.6 ± 9.5	20.6 ± 7.4	23.9 ± 10.1	22.4 ± 10.0	22.5 ± 8.8	21.9 ± 7.7	0.63
Fat (g)	81.9 ± 26.7	79.9 ± 23.1	84.7 ± 31.1	93.6 ± 36.7	86.5 ± 33.9	96.9 ± 38.8	0.256
SFA (g)	26.9 ± 9.2	25.9 ± 9.3	28.0 ± 11.8	30.9 ± 15.0	28.5 ± 10.8	31.5 ± 14.8	0.38
MUFA (g)	35.0 ± 12.6	34.5 ± 11.2	34.5 ± 13.1	41.9 ± 17.2	34.7 ± 11.7	42.8 ± 17.4	0.063
PUFA (g)	12.8 ± 6.7	12.2 ± 4.9	12.8 ± 5.9	11.7 ± 5.0	12.3 ± 6.3	12.4 ± 4.1	0.767
PUFA/SFA	0.4 ± 0.2	0.5 ± 0.2	0.5 ± 0.2	0.4 ± 0.2	0.5 ± 0.2	0.5 ± 0.2	0.272
[PUFA + MUFA]/SFA	1.8 ± 0.4	1.9 ± 0.5	1.8 ± 0.6	1.9 ± 0.5	1.8 ± 0.6	1.9 ± 0.6	0.979
Cholesterol (mg)	330 ± 157	325 ± 149	299 ± 139	287 ± 156	328 ± 128	366 ± 173	0.478
Calcium (mg)	775.3 ± 268.0	727.4 ± 289.6	757.8 ± 246.4	751.6 ± 379.1	687.9 ± 239.4	814.1 ± 372.4	0.062
Iron (mg)	14.9 ± 5.0	12.9 ± 3.2	14.2 ± 4.5	14.4 ± 4.3	13.3 ± 4.0	15.3 ± 5.3	0.041

Data expressed as mean ± SD, *n* = 29. *p* values correspond to the results of the general linear repeated-measures model used to compare the baseline values and end values of GCP, BG and GCP/BG. Carbohydrate (CHO); saturated fatty acids (SFA); monounsaturated fatty acids (MUFA); polyunsaturated fatty acids (PUFA).

**Table 2:** Lipid metabolism biomarkers in serum samples at baseline and at the end of the interventions with the food supplements containing green coffee polyphenols (GCP), beta-glucan (BG) and the mixture of GCP/BG.

	BG	GCP	GCP/BG	*p*
**TG (mg/dL)**				
Baseline	118.4 ± 9.3	119.7 ± 8.5	119.8 ± 9.6	0.906
End	109.0 ± 8.0	118.4 ± 10.5	109.2 ± 8.7	0.942
Rate of change	−0.024 ± 0.051	0.051 ± 0.086	−0.03 ± 0.08	0.785
**TC (mg/dL)**				
Baseline	206.9 ± 6.7	207.0 ± 7.4	206.3 ± 2.3	0.999
End	200.6 ± 5.9	203.2 ± 6.1	203.7 ± 6.9	0.875
Rate of change	−0.023 ± 0.02	−0.008 ± 0.021	−0.01 ± 0.02	0.895
**HDL-C (mg/dL)**				
Baseline	30.0 ± 0.48	30.1 ± 0.64	29.9 ± 1.1	0.980
End	30.1 ± 0.5	29.9 ± 0.6	31.1 ± 1.1	0.997
Rate of change	−0.002 ± 0.002	−0.003 ± 0.003	0.006 ± 0.004	0.177
**LDL-C (mg/dL)**				
Baseline	120.2 ± 5.7	121.0 ± 5.1	122.2 ± 5.3	0.952
End	125.3 ± 5.2	121.5 ± 4.5	125.6 ± 5.7	0.775
Rate of change	0.059 ± 0.0024	0.019 ± 0.03	0.04 ± 0.03	0.733
**VLDL-C (mg/dL)**				
Baseline	23.8 ± 1.9	24.0 ± 1.7	24.0 ± 1.9	0.909
End	21.7 ± 1.6	24.0 ± 1.9	21.5 ± 1.7	0.676
Rate of change	−0.03 ± 0.052 ^a^	0.06 ± 0.08 ^b^	−0.05 ± 0.07 ^a,b^	0.010

Data expressed as mean ± SEM, *n* = 29. *p* values correspond to the results of the linear mixed measures model used to compare the baseline values and end values of GCP, BG and GCP/BG. Rate of change represents [(End value − baseline value)/baseline value]. Triglycerides (TG); total cholesterol (TC); high-density lipoprotein cholesterol (HDL-C); low-density lipoprotein cholesterol (LDL-C); very low-density lipoprotein cholesterol (VLDL-C). ^a,b^ Correspond to differences according to Bonferroni post hoc test.

**Table 3:** Blood pressure and heart rate values at baseline and at the end of the interventions with the food supplements containing green coffee polyphenols (GCP), beta-glucan (BG) and the mixture of GCP/BG.

	BG	GCP	GCP/BG	*p*
**SBP (mmHg)**				
Baseline	124.4 ± 2.3	125.2 ± 2.2	127.4 ± 2.7	0.694
End	121.9 ± 2.4	125.0 ± 2.6	125.6 ± 2.8	0.598
Rate of change	−0.02 ± 0.01	−0.001 ± 0.011	−0.01 ± 0.01	0.178
**DBP (mmHg)**				
Baseline	84.6 ± 1.7	83.3 ± 1.9	83.4 ± 1.7	0.856
End	80.6 ± 1.6	83.7 ± 1.8	82.5 ± 2.0	0.548
Rate of change	−0.05 ± 0.01 ^a^	0.01 ± 0.02 ^b^	−0.01 ± 0.02 ^a,b^	0.027
**Heart Rate (beats/min)**				
Baseline	69.1 ± 1.8	68.7 ± 2.2	69.4 ± 2.0	0.979
End	68.2 ± 1.8	69.7 ± 1.9	70.2 ± 2.2	0.754
Rate of change	−0.01 ± 0.02	0.01 ± 0.02	0.01 ± 0.02	0.637

Data expressed as mean ± SEM, *n* = 29. *p* values correspond to the results of the linear mixed measures model used to compare the baseline values and end values of GCP, BG and GCP/BG. Rate of change represents [(End value − baseline value)/baseline value]. Systolic blood pressure (SBP); diastolic blood pressure (DBP). ^a,b^ Correspond to differences according to Bonferroni post hoc test.

**Table 4:** Glucidic metabolism biomarkers in serum samples at baseline and at the end of the interventions with the food supplements containing green coffee polyphenols (GCP), beta-glucan (BG) and the mixture of GCP/BG.

	BG	GCP	GCP/BG	*p*
**Glucose (mg/dL)**				
Baseline	95.6 ± 2.3	93.7 ± 2.0	95.2 ± 1.7	0.968
End	93.4 ± 1.6	93.2 ± 1.8	93.7 ± 1.6	1.000
Rate of change	−0.015 ± 0.018	−0.002 ± 0.0021	−0.01 ± 0.02	0.464
**Insulin (µUI/mL)**				
Baseline	12.1 ± 1.3	10.5 ± 0.9	10.6 ± 0.9	0.667
End	12.0 ± 1.3	11.4 ± 1.0	11.5 ± 1.1	0.966
Rate of change	0.045 ± 0.061	0.115 ± 0.045	0.14 ± 0.1	0.624
**HbA1c (%)**				
Baseline	5.6 ± 0.1	5.5 ± 0.1.5	5.5 ± 0.1	0.487
End	5.6 ± 0.1	5.6 ± 0.1	5.6 ± 0.1	0.885
Rate of change	0.001 ± 0.006	0.012 ± 0.004	0.01 ± 0.01	0.18
**HbA1c (mmol/mol)**				
Baseline	37.8 ± 0.6	37.0 ± 0.6	37.0 ± 0.6	0.499
End	37.9 ± 0.7	37.7 ± 0.6	37.5 ± 0.6	0.906
Rate of change	0.002 ± 0.009	0.019 ± 0.007	0.02 ± 0.01	0.166

Data expressed as mean ± SEM, *n* = 29. *p* values correspond to the results of the linear mixed measures model use to compare the baseline values and end values of GCP, BG and GCP/BG. Rate of change represents [(End value − baseline value)/baseline value]. Glycosylated haemoglobin (HbA1c).

**Table 5:** Adipsin and adiponectin levels in serum samples at baseline and at the end of the interventions with the food supplements containing green coffee polyphenols (GCP), beta-glucan (BG) and the mixture of GCP/BG.

	BG	GCP	GCP/BG	*p*
**Adipsin (ng/mL)**				
Baseline	992.0 ± 107.2	1051.5 ± 111.0	976.5 ± 121.0	0.655
End	1028.8 ± 97.2	1071.7 ± 111.8	1020.2 ± 116.7	0.895
Rate of change	0.3 ± 0.1	0.01 ± 0.05	0.06 ± 0.05	0.457
**Adiponectin (ng/L)**				
Baseline	58,869.9 ± 7513.3	56,630.0 ± 7456.7	52,758.8 ± 8075.4	0.680
End	51,659.6 ± 7223.2	60,625.1 ± 7584.0	52,582.9 ± 7327.9	0.895
Rate of change	0.1 ± 0.1	0.1 ± 0.1	0.06 ± 0.08	0.646

Data expressed as mean ± SEM, *n* = 29. *p* values correspond to the results of the linear mixed measures model used to compare the baseline values and end values of GCP, BG and GCP/BG. Rate of change represents [(End value − baseline value)/baseline value].

**Table 6:** Serum concentration of hormones related to glucose metabolism at baseline and at the end of the interventions with the food supplements containing green coffee polyphenols (GCP), beta-glucan (BG) and the mixture of GCP/BG.

	BG	GCP	GCP/BG	*p*
**C-peptide (pg/mL)**				
Baseline	1498.0 ± 113.2	1270.2 ± 93.0	1265.6 ± 101.8	0.340
End	1483.0 ± 105.4	1298.7 ± 88.1	1308.1 ± 77.6	0.339
Rate of change	0.01 ± 0.03	0.07 ± 0.06	0.2 ± 0.1	0.966
**Ghrelin (pg/mL)**				
Baseline	482.8 ± 34.6	501.3 ± 42.1	463.3 ± 49.1	0.862
End	559.5 ± 48.5	515.16 ± 41.8	516.9 ± 52.9	0.518
Rate of change	0.19 ± 0.08	0.09 ± 0.07	0.3 ± 0.2	0.765
**GIP (pg/mL)**				
Baseline	162.2 ± 43.95	146.0 ± 42.1	113.7 ± 27.5	0.654
End	165.4 ± 42.1	127.6 ± 34.0	113.8 ± 26.3	0.621
Rate of change	0.17 ± 0.09	0.07 ± 0.11	0.3 ± 0.2	0.433
**GLP-1 (pg/mL)**				
Baseline	191.2 ± 6.8	189.0 ± 9.9	181.8 ± 9.9	0.803
End	195.9 ± 6.0	190.04 ± 8.54	190.0 ± 65.9	0.585
Rate of change	0.04 ± 0.03	0.06 ± 0.06	0.1 ± 0.1	0.433
**Glucagon (pg/mL)**				
Baseline	1774.1 ± 70.4	1746.7 ± 77.6	1744.0 ± 74.7	0.815
End	1832.2 ± 71.2	1785.6 ± 77.15	1760.1 ± 65.9	0.585
Rate of change	0.04 ± 0.02	0.04 ± 0.03	0.00 ± 0.00	0.413
**Insulin (pg/mL)**				
Baseline	478.2 ± 55.14	363.3 ± 38.6	374.1 ± 45.7	0.338
End	468.4 ± 49.6	378.4 ± 38.8	375.6 ± 35.3	0.273
Rate of change	0.04 ± 0.06	0.15 ± 0.1	0.7 ± 0.5	0.669
**Leptin (pg/mL)**				
Baseline	5296.9 ± 592.8	4745.0 ± 581.7	4945.9 ± 618.2	0.920
End	5778.8 ± 613.8	4782.62 ± 507.44	4813.1 ± 609.6	0.669
Rate of change	0.11 ± 0.04	0.11 ± 0.07	0.10 ± 0.09	0.668
**PAI (pg/mL)**				
Baseline	45,419.5 ± 3251.7	45,570.0 ± 3273.5	39,554.5 ± 3529.0	0.339
End	49,245.5 ± 3642.2	44,975.74 ± 3241.79	44,377.9 ± 3940.7	0.631
Rate of change	0.18 ± 0.13	0.16 ± 0.14	0.20 ± 0.10	0.927
**Resistin (pg/mL)**				
Baseline	6783.9 ± 444.07	7196.6 ± 688.9	5752.6 ± 676.7	0.141
End	7860.6 ± 612.0	6652.4 ± 737.5	6825.9 ± 632.3	0.590
Rate of change	0.19 ± 0.07	0.16 ± 0.17	1.0 ± 0.5	0.803
**Visfatin (pg/mL)**				
Baseline	8160.6 ± 572.9	8241.2 ± 875.8	7857.1 ± 822.8	0.951
End	8206.5 ± 530.3	7784.9 ± 645.1	7633.7 ± 626.2	0.900
Rate of change	0.03 ± 0.04	0.05 ± 0.09	0.10 ± 0.09	0.803

Data expressed as mean ± SEM, *n* = 29. *p* values correspond to the results of the linear mixed measures model used to compare the baseline values and end values of GCP, BG and GCP/BG. Rate of change represents [(End value − baseline value)/baseline value]. Gastric inhibitory polypeptide (GIP); glucagon-like peptide-1 (GLP-1); plasminogen activator inhibitor (PAI).

**Table 7:** ASAT, ALAT and hsPCR concentrations in serum samples at baseline and at the end of the interventions with the food supplements containing green coffee polyphenols (GCP), beta-glucan (BG) and the mixture of GCP/BG.

	BG	GCP	GCP/BG	*p*
**ASAT (UI/I)**				
Baseline	23.0 ± 1.6	23.5 ± 1.4	22.7 ± 1.1	0.824
End	23.3 ± 1.1	23.8 ± 0.8	25.0 ± 1.2	0.641
Rate of change	0.065 ± 0.042	0.061 ± 0.04	0.15 ± 0.07	0.352
**ALAT (UI/I)**				
Baseline	25.9 ± 1.9	28.1 ± 2.3	28.7 ± 2.3	0.615
End	29.9 ± 2.5	30.5 ± 2.4	31.1 ± 2.4	0.866
Rate of change	0.199 ± 0.094	0.139 ± 0.061	0.17 ± 0.08	0.983
**hsPCR (mg/dL)**				
Baseline	0.2 ± 0.03	0.18 ± 0.04	0.22 ± 0.05	0.965
End	0.14 ± 0.03	0.15 ± 0.03	0.16 ± 0.03	0.815
Rate of change	0.0006 ± 0.11	0.01 ± 0.08	−0.06 ± 0.11	0.434

Data expressed as mean ± SEM, *n* = 29. *p* values correspond to the results of the linear mixed measures model used to compare the baseline values and end values of GCP, BG and GCP/BG. Rate of change represents [(End value − baseline value)/baseline value]. Aspartate amino transferase (ASAT); alanine amino transferase (ALAT); high-sensitivity C-reactive protein (hsPCR).

## Data Availability

Data may be available from the corresponding author upon request.
